# Identification of *Salmonella* Bredeney Resistant to Third-Generation Cephalosporins in Saudi Arabia

**DOI:** 10.3389/fcimb.2019.00390

**Published:** 2019-11-20

**Authors:** Ayman Ahmad Al kraiem, Yingchun Zeng, Xixiang Huo, Kun Yang, Fahd Al kraiem, Jingliang Qin, Yujun Cui, Biao Kan, Meiying Yan, Guang Yang, Tie Chen

**Affiliations:** ^1^Department of Clinical Immunology, Tongji Medical College, Tongji Hospital, Huazhong University of Sciences and Technology, Wuhan, China; ^2^Department of Biomedical Engineering, College of Life Science and Technology, Huazhong University of Science and Technology, Wuhan, China; ^3^Department of Biology, College of Science, Taibah University, Medina, Saudi Arabia; ^4^Hubei Provincial Center for Disease Control and Prevention (CDC), Wuhan, China; ^5^Department of Pathogen Biology and Immunology, Shihezi University School of Medicine, Shihezi, China; ^6^Pilgrims City Hospital, Ministry of Health, Medina, Saudi Arabia; ^7^State Key Laboratory of Pathogen and Biosecurity, Beijing Institute of Microbiology and Epidemiology, Beijing, China; ^8^School of Basic Medical Sciences, Anhui Medical University, Hefei, China; ^9^Chinese Center for Disease Control and Prevention, National Institute for Communicable Diseases Control and Prevention, Beijing, China

**Keywords:** *Salmonella enterica*, Bredeney serotype, antimicrobial resistance, cephalosporins, Saudi Arabia

## Abstract

The rapidly increasing prevalence and spread of antibiotic-resistant *Salmonella* worldwide have become a thorny problem that poses a serious threat to human health. It is speculated that antibiotic abuse, frequent traveling, and mass gatherings accelerate this threat. To explore this hypothesis, we investigated 13 *Salmonella* isolates from Medina, Saudi Arabia and 15 from China as the control group using typical methods of serotype identification, antibiotic resistance tests, pulsed-field gel electrophoresis (PFGE), and multi-locus sequence typing (MLST). Our results indicated that the isolates from China showed greater serotype diversity and a higher antimicrobial resistance rate, which was consistent with results from other studies in China. In contrast, the Saudi Arabian isolates were mainly identified as Serovar Bredeney and were resistant to a limited number of antibiotics. Interestingly, two of the Bredeney isolates was resistant to third-generation cephalosporins but sensitive to all other tested antibiotics. To confirm the results and understand the underlying molecular mechanisms of these isolates, whole-genome sequencing (WGS) was performed. We discovered that several cephalosporin resistance-associated genes were shared with other strains, but one gene (LEN-23) was unique. Therefore, to the best of our knowledge, we concluded that this study is the first to report the emergence of *Salmonella* Bredeney resistant to third-generation cephalosporins in Saudi Arabia.

## Introduction

The emergence of antibiotic-resistant microbes has become a major problem; the inexorable rise of new resistant isolates has been widely reported, outpacing the rate of replacement of obsolete antibiotics with new effective ones. The interventions for reducing the spread of resistance are currently ineffective, and the risk of spreading these resistant microbes increases tremendously when crowds attend mass gatherings. The most prevalent antibiotic-resistant microbe is *Salmonella*.

*Salmonella* is a gram-negative, motile, non-spore forming, facultative anaerobic, rod-shaped bacterium belonging to the family Enterobacteriaceae (Issenhuth-Jeanjean et al., 2014). Globally, *Salmonella* is considered a primary cause of foodborne illnesses, such as typhoid fever, paratyphoid fever, and food poisoning. Salmonellosis is a major cause of gastroenteritis in both developed and developing countries, which leads to high morbidity and economic burden (Yoshida et al., 2014; Al kraiem et al., 2018).

The taxonomic classification of *Salmonella* has continuously changed over time. The genus *Salmonella* is classified into *S. bongori* and *S. enterica*. *S. enterica* is further divided into six subspecies: *S. enteric* subsp. *enterica, S. enterica* subsp. *salamae, S. enterica* subsp. *arizonae, S. enterica* subsp. *diarizonae, S. enterica* subsp. *houtenae*, and *S. enterica* subsp. *Indica*. Based on phenotypic and genotypic studies (Achtman et al., 2012; Issenhuth-Jeanjean et al., 2014; Smith et al., 2016), *Salmonella* is categorized into typhoidal and non-typhoidal *Salmonella* (NTS) (Marzel et al., 2016), with the latter estimated to cause 93.8 million cases of gastroenteritis and 155,000 mortalities worldwide (Yoshida et al., 2014; Al kraiem et al., 2018).

The emergence of multidrug-resistant NTS strains has been reported all over the world (Ayed, 2014; Marks et al., 2017; Mohan et al., 2019), which is assumed to be due to the misuse of antibiotics in both humans and domestic livestock. It is a general practice of doctors to prescribe third-generation cephalosporins to whoever shows signs of diarrhea or infection in the city of Medina, near the Mecca area of Saudi Arabia (personal communications). In addition, the Hajj, an annual Muslim pilgrimage to Mecca, Saudi Arabia, is one of the largest religious mass gatherings in the world, comprising about two million pilgrims from 185 countries (Memish et al., 2014). As part of the Hajj rituals, pilgrims visit various sacred places around the city of Mecca. Most of them also travel to the city of Medina to visit the second holiest site of Islam, the Prophet's mosque containing the tomb of the Prophet Muhammad (Hoang and Gautret, 2018).

It is generally believed that the emergence of multidrug-resistant NTS strains is due to rise in antibiotic abuse, frequent travel, and mass gatherings. Therefore, this study investigated the antimicrobial susceptibility profile of *Salmonella* strains isolated from the city of Medina, in the Mecca area of Saudi Arabia.

## Materials and Methods

### *Salmonella* Isolates

Twenty-eight *Salmonella* clinical isolates, consisting of 13 isolates obtained from the Regional Laboratory Center, Medina, Saudi Arabia and 15 isolates from the China Center for Disease Control and Prevention (CDC) in Beijing, China, were identified using standard biochemical tests and serotyped by agglutination tests according to the White-Kauffmann-Le Minor scheme at the Hubei Provincial Center for Disease Control and Prevention.

### Antimicrobial Susceptibility Profiles

The susceptibility of *Salmonella* isolates to ciprofloxacin, cefoxitin, cefotaxime, gentamicin, trimethoprim/sulfamethoxazole, chloramphenicol, nalidixic acid, and tetracycline were determined using the 96-well Sensititre™ MIC panels (Sensititre AIM™ Automated Inoculation Delivery System, Thermo Fisher Scientific Inc., USA). Meanwhile, susceptibility to ampicillin (10 μg), ceftriaxone (30 μg), levofloxacin (5 μg), cefixime (5 μg), and kanamycin (30 μg) (STAR, Beijing Tiantan Pharmaceutical Biotechnology Development Company) was determined using the Kirby-Bauer disk diffusion method. Furthermore, the minimum inhibitory concentration (MIC) of the third-generation cephalosporins (ceftriaxone and cefixime) were determined through the multi-proportion dilution test. Briefly, agar plates were prepared containing gradient antibiotic concentrations (from 128 to 0.5 μg/ml). Bacterial suspensions were then added to the ceftriaxone and cefixime plates, respectively. Strains with MIC of 0–5, 6–15, and ≥16 μg/ml were defined as low, medium, and high, respectively, in terms of ceftriaxone-resistance, and those with MIC of 0–8, 9–18, and ≥18 μg/ml were defined as low, medium and high, respectively, in terms of cefixime-resistance. *Escherichia coli* ATCC 25922 and *Salmonella typhimurium* LT2 were used as quality reference isolates for all tests. The results of the MIC test and disk diffusion were interpreted according to the criteria set by the Clinical and Laboratory Standards Institute (CLSI, 2013).

### Pulsed-Field Gel Electrophoresis (PFGE)

PFGE of 28 isolates was performed following the PulseNet standardized protocol (www.cdc.gov/pulsenet). Genomic DNA fragments were prepared from each isolate with the restriction enzyme XbaI (NEB), incubated at 37°C for 2 h. Macrorestriction fragments were run on a 1% agarose gel in 0.5 × TBE buffer (containing 50 μM thiourea to prevent DNA degradation) in a CHEF Mapper PFGE system (BioRad). Gels were stained with ethidium bromide and photographed with UV transillumination. *Salmonella* Braenderup H9812 XbaI-digested DNA was used as the molecular weight standard. The settings used were: 6 V/cm, an angle of 120°, initial switch time of 2.16 s, final switch time of 63.8 s, and a run time of 19.5 h.

Macrorestriction fragments were compared using the fingerprint analysis module of the BioNumerics software (Version 7.6, Applied Math). The isolates were considered dissimilar if one or more DNA bands appeared to have different molecular weights. The similarity index was calculated using the Dice similarity coefficient, and a similarity dendrogram was constructed using the unweighted pair group method using average linkages (UPGMA) with 1% optimization and 1% band position tolerance.

### Multi-Locus Sequence Typing (MLST)

Multi-locus sequence typing was carried out using the Warwick University MLST protocols and database. Briefly, all *Salmonella* isolates were grown overnight in Luria-Bertani (LB) medium at 37°C. Total genomic DNA was extracted using a TIANamp bacteria DNA kit (TianGen DNA Kit DP302, Beijing, China) according to the manufacturer's instructions. To characterize these isolates, seven fragments of conserved housekeeping genes (*aroC, dnaN, hemD, hisD, purE, sucA*, and *thrA*) with known chromosomal positions and functions were amplified using specific primer sets in the Warwick University MLST database (http://mlst.warwick.ac.uk/mlst/dbs/Senterica/documents/~primersEnterica_html).

The PCR cycling conditions for *thrA, sucA, aroC*, and *hemD* were as follows: initial denaturation (95°C for 10 min), followed by 35 cycles of denaturation (94°C for 30 s), annealing (55°C for 30 s), and elongation (72°C for 30 s), and a final elongation step (72°C for 5 min). The rest of the genes had similar conditions except for the annealing temperature (50°C for 30 s) and elongation time (1 min). The PCR products were transferred to agarose gel (1.5%) and visualized with ethidium bromide staining and UV illumination (Beijing Liuyi Biotechnology Co. LTD, Beijing, China).

The PCR products (which included the DNA amplicons and primers used) were purified using the Mag-bind PCR Purification Kit (Icongene Biotech Company, Wuhan, China) and then sent for sequencing at the Icongene Biotech Company (Wuhan, China). The chromatograms obtained from the seven housekeeping genes were compared with the *Salmonella* database in the MLST database and analyzed with BioNumerics version 7.6 (Applied Maths).

### Primers for Resistant (R) Plasmids

To identify the mechanism underlying cephalosporin resistance, the existence of the R-plasmid was detected using two pairs of R-plasmid primers; R-PLASMID-F1, CAAGGTACCGTAACCACCCC; R-PLASMID-R1, CCCGATGATGGCATAAGGCT; R-PLASMID-F2, CCCACCGTGACGAAGATTCA; R-PLASMID-R2, ACCTGAACGATCCGCAATGT.

### Whole-Genome Sequencing (WGS) and Phylogenetic Analysis

WGS was performed on two isolates (*Salmonella* spp. B3 and STC2) on an Illumina MiSeq platform with 2 × 150 bp paired-end library. Sequencing reads were used in SPAdes (http://cab.spbu.ru/software/spades/) for draft genome assembly. After assembly, genome sizes of 4.6 and 5.1 Mb were obtained for the two isolates, respectively. SNP identification: The two assemblies were aligned against a reference genome CVM19633 (accession: NC_011094) using MUMmer to generate the whole genome alignments and identify SNPs in the core genome. Raw sequencing reads were mapped to the assemblies to evaluate the SNP accuracy using SOAPaligner. The SNPs located in repetitive regions and with low sequence quality (quality score <20 or containing more than five consecutive *N* reads) were filtered out as previously described (Cui et al., 2013). After filtering, 327,505 SNPs in total were identified from the 603 isolates, which were used in the neighbor-joining tree (NJ tree) construction using TreeBeST 1.9.2 (http://treesoft.sourceforge.net/treebest.shtml). The phylogenetic tree was visualized using FigTree (http://tree.bio.ed.ac.uk/software/figtree/). SNP annotation: 4,048 SNP sites were mapped to the reference genome (the closest strain; *Salmonella enterica* subsp. *enterica serovar* Bredeney str. CFSAN001080) and these SNPs were annotated based on reference genome annotation information.

## Results

### Strains Resistant to Third-Generation Cephalosporins Was Detected

Two methods (disk diffusion and MIC) were applied in this study to determine the susceptibility of these 28 strains to 13 antibiotics (Figure 2 and Supplementary Table 2). The results showed that all the isolates were susceptible to cefotaxime, gentamicin, levofloxacin, and kanamycin. Aside from these antibiotics, all the isolates from Saudi Arabia were susceptible to ciprofloxacin, cefoxitin, trimethoprim/sulfamethoxazole, nalidixic acid, tetracycline, ampicillin, and levofloxacin. Nine isolates from Saudi Arabia and three from China had intermediate resistance to chloramphenicol, and five from China had intermediate resistance to ciprofloxacin. Among the isolates from China, four showed antimicrobial resistance to tetracycline, one to cefoxitin, five to nalidixic acid, and one to trimethoprim/sulfamethoxazole and ampicillin. Interestedly, all isolates from China were susceptible to third-generation cephalosporins (i.e., ceftriaxone and cefixime), whereas two isolates from Saudi Arabia were resistant but susceptible, to all other tested antibiotics.

### Most Strains Isolated From Saudi Arabia Could Not Be Serotyped Through Conventional Methods

**Figure 1:**
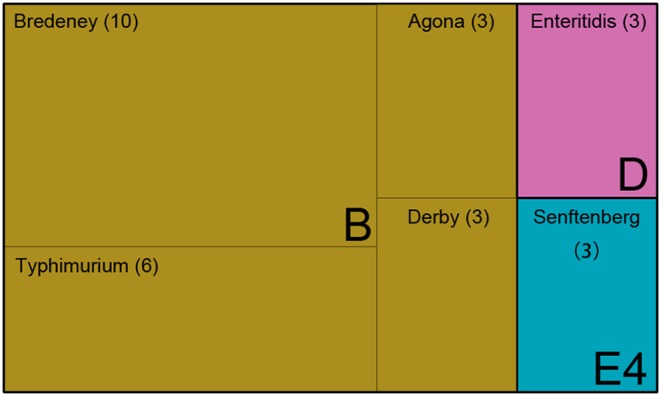
The serotype of 28 *Salmonella* isolates. Treemap showing 28 isolates identified as serogroups B, D, and E, and subclassified as serovar Bredeney, Typhimurium, Agona, Derby, Enteritidis, and Senftenberg.

The *Salmonella* isolates were serotyped by agglutination tests according to the White-Kauffmann-Le Minor scheme. The isolates were identified as serogroups B, D, and E4. Further, these isolates were subgrouped as *S. enterica* Typhimurium (*n* = 6), *S*. *enterica* Agona (*n* = 3), *S*. *enterica* Derby (*n* = 3), *S*. *enterica* Enteritidis (*n* = 3), and *S*. *enterica* Senftenberg (*n* = 3) (Figure 1 and Supplementary Table 1). However, 10 isolates of serogroup B were unable to be typed by agglutination tests, indicating that the rare serovars were possibly included in the Saudi isolates.

### Ten Strains Isolated From Saudi Arabia Were Detected as the Bredeney Serovar by PFGE Analysis

**Figure 2:**
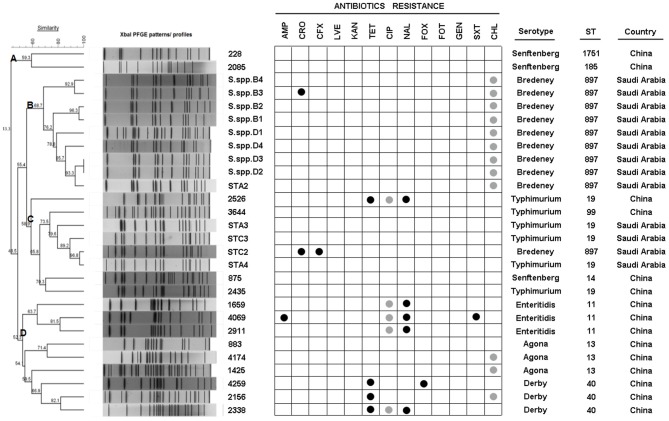
PFGE XbaI patterns, ID, antibiotic-resistance, serotype, sequence type (ST), and origin of 28 isolates. Dendrogram showing the cluster analysis of different PFGE XbaI patterns from 28 isolates generated by Bionumerics software using UPGMA methods. Antibiotic-resistance was detected by MIC and disk diffusion. Serotypes were detected using agglutination tests. STs were determined by MLST.

As indicated above, several isolates could not be serotyped by agglutination tests. The *Salmonella* isolates were then sub-typed using PFGE into 27 XbaI macrorestriction patterns (Figure 2). The range of PFGE restriction fragments that were used for analysis was 20–1,135 kb. The number of restriction fragments produced by XbaI ranged from 13 to 30. Strains of serovar Agona, Senftenberg, and Derby exhibited only three patterns, whereas Typhimurium and Bredeney showed six and nine different patterns, respectively. Corresponding to the homology, the strains were clustered into four groups (A, B, C, and D). The highest similarity of patterns was observed in cluster B (68.7%), which contains the Bredeney serovar, whereas the lowest similarity was noticed in cluster D (52.3%), containing three serovars (Enteritidis, Agona, and Derby). In summary, ten isolates from Saudi were indicated as Bredeney serovars.

### Identification of Clonal Fragments in Bredeney Serovars With MLST

**Figure 3:**
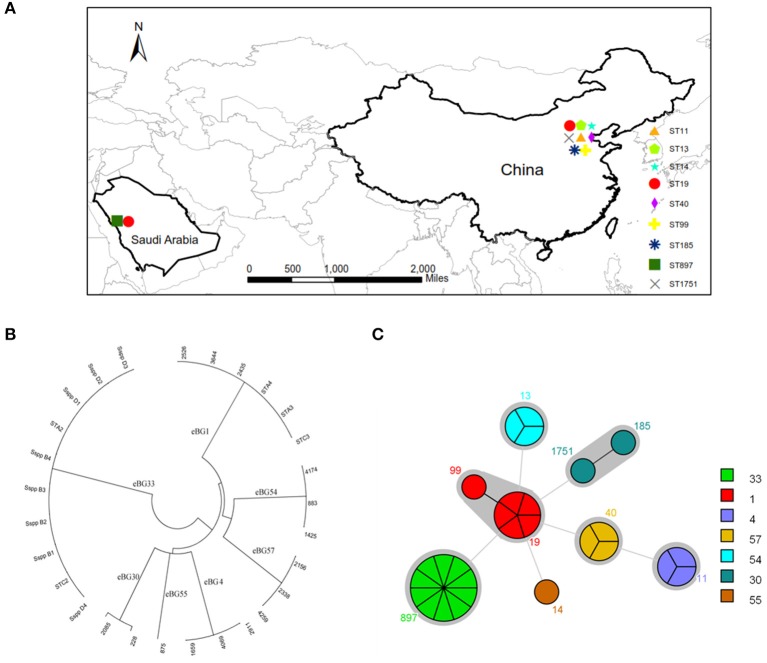
MLST analysis of 28 isolates. **(A)** The distribution of STs. **(B)** clonal-complex (CC) (1, 4, 30, 33, 54, 55, and 57) were identified by eBURST analysis. **(C)** STs and CC.

The question now was whether the 10 Bredeney serovars were from a single source or multiple sources. MLST was conducted to determine the nucleotide polymorphisms within seven defined housekeeping genes (*aroC, dnaN, hemD, hisD, purE, sucA*, and *thrA*). The results of all *Salmonella* isolates were identified and compared using the *Salmonella* MLST database (http://mlst.warwick.ac.uk/mlst/dbs/Senterica/documents/primersEnterica_html). Among the 28 strains, nine sequence types (STs) were identified by their seven-gene MLST patterns, as follows: two STs of *S*. Typhimurium (ST 19 and ST 99), three STs of *S*. Senftenberg (ST 14, ST 1751, and ST 185), and one ST for *S*. Agona (ST 13), *S*. Derby (ST 40), *S*. Enteritidis (ST 11), and *S*. Bredeney (ST 897) (Figure 2). The isolates from China showed greater serotype diversity with eight STs while the Saudi Arabian ones had only two STs (Figure 3A). Ten isolates (36%) created the largest PFGE cluster and were identified as ST897 belonging to the Bredeney serovar. Six isolates (21%) were typed as ST19 and ST 99 and belonged to *S*. Typhimurium. The allelic profile of ST19 was 10-7-12-9-5-9-2, and the allelic profile of ST99 was 10-7-12-9-5-9-46. All PFGE patterns in cluster A belonged to *S*. Senftenberg, while cluster B patterns were typed as ST897, belonging to *S*. Bredeney. Cluster C consisted of three different types of serovars: *S*. Typhimurium, *S*. Senftenberg, and *S*. Bredeney. Lastly, cluster D comprised *S*. Agona, *S*. Derby, and *S*. Enteritidis (Figure 2). Therefore, the results confirm the results acquired from PFGE that 10 isolates from Saudi Arabia belong to the Bredeney serovars.

To support our previous findings and analysis, MLST technologies were used to compare the clonal-complex (CC) to clustering results from Achtman et al. (2012); Murgia et al. (2015). Seven CCs (1, 4, 30, 33, 54, 55, and 57) were identified by eBURST analysis (Figure 3B). CC1 comprised two STs, namely ST19 and ST99, belonging to *S*. Typhimurium. CC4 consisted of ST11 belonging to *S*. Enteritidis. CC30 comprised ST185 and ST1751, belonging to *S*. Senftenberg. CC33 comprised ST897, belonging to *S*. Bredeney. To the best of our knowledge, this is the first report on the occurrence of *S*. Bredeney in Saudi Arabia. CC54 comprised ST13, CC55 comprised ST14, and CC57 comprised ST40, belonging to serovars Agona, Senftenberg, and Derby, respectively (Figure 3C).

Furthermore, the combined examination of all three methods revealed interesting associations among antimicrobial susceptibility, pulsed-field fingerprints, and MLST. For example, cluster A was made up of isolates that were susceptible to all tested antimicrobials and comprised *S*. Senftenberg strains with ST1751 and ST185 under the same clonal-complex, CC55. These two STs are double-locus variants at the *hemD* and *purE* locus. Cluster B, which was identified as *S*. Bredeney, originated from Saudi Arabia, exhibited intermediate chloramphenicol-resistance, and identified as ST897. Cluster C contained almost-susceptible isolates, except for two isolates (ST19 and ST897). The major sequence types of cluster C were ST19 and ST99, which are single-locus variants at the *thrA* locus. Cluster D was made up of three sequence types (ST11, ST13, and ST40), which had completely different sequences at the seven loci, and differed in their antimicrobial susceptibility. ST11 (CC4) was composed of nalidixic-resistance strains, whereas ST13 (CC54) and ST40 (CC57) contained almost-susceptible and tetracycline-resistant strains, respectively (Figures 2, 3C).

In summary, three significant results were obtained from the MLST studies: (1) the results confirmed those acquired from PFGE, that ten isolates from Saudi Arabia belong to the Bredeney serovars. (2) To our knowledge, this is the first report of the occurrence of *S*. Bredeney in Saudi Arabia. (3) New clusters of antimicrobial susceptibility of *S*. Bredeney were detected.

### Cephalosporin Resistance-Associated Genes

First, the existence of R-plasmids was not detected in the isolates of *Salmonella* spp. B3 and STC2 using the two R-plasmid primers listed in the Methods section.

To identify the underlying mechanism of cephalosporin resistance, the whole genome sequencing of isolates *Salmonella* spp. B3 and STC2 were performed, as well as a comparative genomics analysis with 601 public whole genomes (Supplementary Table 3).

A total of 327,505 genome-wide SNPs was identified from 603 genomes of *S*. Enteritidis. To explore the relationships of the isolates, a neighbor-joining (NJ) tree was constructed based on concatenated SNPs. It was found that the closest related strain of the two new sequenced isolates was *Salmonella enterica* subsp. *enterica* serovar Bredeney str. CFSAN001080; hence, the serotype of the closest strain was consistent with that of the two isolates. The phylogenetic tree is shown in Supplementary Figure 1 (the two isolates are shown in red). In total, 4,048 SNPs were identified between the closest strain and isolates (three strains); the number of SNPs between the two samples is 0.

The Comprehensive Antibiotic Resistance Database (CARD, a bioinformatics database of resistance genes, their products, and associated phenotypes.) was used for drug resistance annotation of the strains.

CARD resistance analysis (https://card.mcmaster.ca/home) was performed on *Salmonella* spp. B3, STC2, and three closely related strains (indicated with the blue font in Supplementary Figure 1). The results show that *Salmonella* spp. B3 and STC2 shared 13 cephalosporin resistance-associated genes (Table 1 and Supplementary Tables 3–8). The first row in Table 2 represents the drug resistance genes of *Salmonella* spp. B3 and STC2, the first column represents the different strains, and cyan indicates the genes that were detected in these strains (Table 2). From the sequencing analysis, *Salmonella* Bredeney was identified as resistant to third-generation cephalosporins in Saudi Arabia.

**Table 1:** Cephalosporin-resistant genes shared by *Salmonella* spp. B3 and STC2.

**Resistance gene**	**Drug class**	**Resistance mechanism**
acrB	Fluoroquinolone antibiotic; cephalosporin; tetracycline antibiotic; rifamycin antibiotic; penam; phenicol antibiotic; triclosan; glycylcycline	Antibiotic efflux
*Escherichia coli* acrA	Rifamycin antibiotic; fluoroquinolone antibiotic; tetracycline antibiotic; penam; phenicol antibiotic; glycylcycline; triclosan; cephalosporin	Antibiotic efflux
*Escherichia coli* acrR with mutation conferring multidrug antibiotic resistance	Rifamycin antibiotic; fluoroquinolone antibiotic; tetracycline antibiotic; penam; phenicol antibiotic; glycylcycline; triclosan; cephalosporin	Antibiotic efflux; antibiotic target alteration
*Escherichia coli* marR mutant conferring antibiotic resistance	Rifamycin antibiotic; fluoroquinolone antibiotic; tetracycline antibiotic; penam; phenicol antibiotic; glycylcycline; triclosan; cephalosporin	antibiotic efflux; antibiotic target alteration
*Escherichia coli* soxR with mutation conferring antibiotic resistance	Rifamycin antibiotic; penam; fluoroquinolone antibiotic; tetracycline antibiotic; triclosan; cephalosporin; phenicol antibiotic; glycylcycline	Antibiotic efflux; antibiotic target alteration
*Escherichia coli* soxS with mutation conferring antibiotic resistance	Rifamycin antibiotic; penam; fluoroquinolone antibiotic; carbapenem; tetracycline antibiotic; cephamycin; triclosan; cephalosporin; phenicol antibiotic; penem; monobactam; glycylcycline	Antibiotic efflux; reduced permeability to antibiotic; antibiotic target alteration
golS	Phenicol antibiotic; carbapenem; penam; penem; monobactam; cephamycin; cephalosporin	Antibiotic efflux
Haemophilus influenzae PBP3 conferring resistance to beta-lactam antibiotics	Cephamycin; carbapenem; penam; cephalosporin; monobactam	Antibiotic target alteration
marA	Rifamycin antibiotic; penam; fluoroquinolone antibiotic; carbapenem; tetracycline antibiotic; cephamycin; triclosan; cephalosporin; phenicol antibiotic; penem; monobactam; glycylcycline	Antibiotic efflux; reduced permeability to antibiotic
mdsA	Phenicol antibiotic; cephamycin; penam; carbapenem; penem; cephalosporin; monobactam	Antibiotic efflux
mdsC	Phenicol antibiotic; cephamycin; penam; carbapenem; penem; cephalosporin; monobactam	Antibiotic efflux
sdiA	Rifamycin antibiotic; fluoroquinolone antibiotic; tetracycline antibiotic; penam; phenicol antibiotic; glycylcycline; triclosan; cephalosporin	Antibiotic efflux
TEM-60	Penam; penem; cephalosporin; monobactam	Antibiotic inactivation

**Table 2:** Specific resistant genes of *Salmonella* spp. B3 and STC2.

**Isolates**	**LEN-21**	**LEN-23**	**vgaC**	**LEN-26**	**OKP-B-20**
*Salmonella* spp. B3*					
*Salmonella* spp. STC2					
*S. enterica* serovar Give CFSAN024229					
*S. enterica* serovar Bredeney str.CFSAN001080					
*S. enterica* serovar Bredeney NCTC6026					

Cyan indicates that the particular gene was detected. *The specific resistance gene of Salmonella spp. B3 is LEN-23, which is related to penam- and penem-resistance.

## Discussion

The occurrence of antimicrobial-resistant *Salmonella* in Saudi Arabia has been reported by several studies. Somily et al., collected 213 *Salmonella* isolates which were mostly non-typhi serotypes between January 2007 and May 2009 at King Khalid University Hospital in Riyadh, Saudi Arabia, and they reported that significantly higher proportions of *Salmonella* were resistant to nalidixic acid (46%) (Somily et al., 2012). Elhadi et al. analyzed NTS strains from 158 stool specimens of patients in the Eastern Province of Saudi Arabia from September 2008 to April 2011 and reported that the resistance to ampicillin was the most prevalent (31.3%) (Elhadi et al., 2013). El-Tayeb et al. collected 33 *S. enterica* isolates from clinical samples at King Khalid University Hospital and a sewage treatment plant in Riyadh, Saudi Arabia, and reported that 87.9–90.9% of isolates were resistant to 1st and 2nd generation cephalosporins (El-Tayeb et al., 2017).

Although the aforementioned data seem to be sourced from different areas at various times with inconsistent antimicrobial resistance, the pool of isolates was in fact procured from a single center, which might not constitute a representative epidemiological sample of the country. Therefore, data from other areas of Saudi Arabia are required to improve our understanding of antimicrobial resistance dynamics.

In the current study, only 13 isolates were collected from the patients in Medina, Saudi Arabia; however, *Salmonella* Bredeney was found to be the most prevalent. Among these isolates, two were resistant to third-generation cephalosporins as they harbored 13 resistance-associated genes. Although *Salmonella* isolates resistant to third-generation cephalosporins with overall resistance rates of 3, 14.93, and 6%, respectively, have been reported in previous studies (Somily et al., 2012; Elhadi et al., 2013; El-Tayeb et al., 2017), they were identified as other subspecies, such as serotype D1 and *S*. Arizonae. Therefore, the present study is the first to report the emergence of *S*. Bredeney resistant to third-generation cephalosporins in Saudi Arabia.

Some isolates cannot be typed using the recommended sera due to the limitations of the conventional serotype method. Somily et al. were unable to serotype 23 isolates (Somily et al., 2012), and El-Tayeb et al. identified four unserotypable isolates by using the VITEK 2-C15 identification system (El-Tayeb et al., 2017). In the present study, to accurately identify the isolates, both the conventional and advanced methods were used. Ten isolates of serogroup B were unable to be typed by agglutination tests and were alternatively examined by MLST profiling, which subsequently identified them as *S. enterica* Bredeney. Further, the underlying mechanism of resistance was revealed with whole-genome sequencing. Because each of these methods has its advantages and disadvantages, their combination can facilitate accurate identification and the acquisition of comprehensive information.

This study has certain limitations. First, all Saudi Arabian *Salmonella* spp. were isolated from patients in Medina; thus, the prevalence of antibiotic-resistant *Salmonella* is still unknown. Second, the sample size was relatively small. Despite these limitations, we isolated a rare *Salmonella* serotype that was resistant to third-generation cephalosporins and also elucidated the mechanism underlying its resistance using WGS.

In summary, the initial goal of this study was to examine and analyze the antimicrobial susceptibility of 13 *Salmonella enterica* subsp. *enterica* isolated from Medina, Saudi Arabia. However, two isolates of *S*. Bredeney were identified in this study for the first time that were resistant to third-generation cephalosporin, and a possible mechanism of resistance was elucidated. The data obtained from this study may alert the policy-makers to the emergence of *S*. bredeney resistant to third-generation cephalosporin in Saudi Arabia. In the future, large-scale studies should be performed to acquire information to construct standardized regional and national antimicrobial resistance surveillance data systems in Saudi Arabia is recommended.

## Data Availability Statement

The raw data supporting the conclusions of this manuscript will be made available by the authors, without undue reservation, to any qualified researcher.

## Author Contributions

All authors contributed to the conception and design of experiments. They all also participated in manuscript writing, revision, and approval for final submission.

### Conflict of Interest

The authors declare that the research was conducted in the absence of any commercial or financial relationships that could be construed as a potential conflict of interest.
