# Supplementary material for: Identification of Salmonella Bredeney Resistant to Third-Generation Cephalosporins in Saudi Arabia
Source: Front Cell Infect Microbiol. 2019 Nov 20;9:390. doi: 10.3389/fcimb.2019.00390 (PMC6879462; doi:10.3389/fcimb.2019.00390)
Supplement: Supplementary file 1 [file Table_1.doc]

**Supplementary Table 1 Serotyping and serogrouping *of Salmonella* isolates.**

| ***Salmonella* serotype (serovar)** | **Serogroup^a^** | **Isolates ID** |
| --- | --- | --- |
| Senftenberg | E4 | 228 |
| Senftenberg | E4 | 875 |
| Agona | B | 883 |
| Agona | B | 1425 |
| Enteritidis | D | 1659 |
| Senftenberg | E4 | 2085 |
| Derby | B | 2156 |
| Agona | B | 2338 |
| Typhimurium | B | 2435 |
| Typhimurium | B | 2526 |
| Enteritidis | D | 2911 |
| Typhimurium | B | 3644 |
| Enteritidis | D | 4069 |
| Derby | B | 4174 |
| Derby | B | 4259 |
| Bredeney  **^b^** | B | STC2 |
| Typhimurium | B | STC3 |
| Bredeney  **^b^** | B | S.spp.B1 |
| Bredeney  **^b^** | B | S.spp.B2 |
| Bredeney  **^b^** | B | S.spp.B3 |
| Bredeney  **^b^** | B | S.spp.B4 |
| Bredeney  **^b^** | B | STA2 |
| Typhimurium | B | STA3 |
| Typhimurium | B | STA4 |
| Bredeney  **^b^** | B | S.spp.D1 |
| Bredeney  **^b^** | B | S.spp.D2 |
| Bredeney  **^b^** | B | S.spp.D3 |
| Bredeney  **^b^** | B | S.spp.D4 |

^a^Serogroup determined according to Kauffman-White-Le Minor scheme.

^b^Serovar inferred by MLST profile.
