# Supplementary material for: Identification of Salmonella Bredeney Resistant to Third-Generation Cephalosporins in Saudi Arabia
Source: Front Cell Infect Microbiol. 2019 Nov 20;9:390. doi: 10.3389/fcimb.2019.00390 (PMC6879462; doi:10.3389/fcimb.2019.00390)
Supplement: Supplementary file 2 [file Table_2.docx]

**Supplementary Table 2. Antimicrobial susceptibility profiles**

**Disc diffusion - Zone inhibition mm（susceptibility result):**

| **KAN** | **CFX** | **LVE** | **CRO** | **AMP** | *Salmonella* isolates |
| --- | --- | --- | --- | --- | --- |
| 29  (S) | 0  (R) | 35  (S) | 0  (R) | 31  (S) | STC2 |
| 26  (S) | 31  (S) | 35  (S) | 31  (S) | 28  (S) | STC3 |
| 24  (S) | 33  (S) | 35  (S) | 36  (S) | 22  (S) | S.spp.B1 |
| 23  (S) | 31  (S) | 32  (S) | 32  (S) | 25  (S) | S.spp.B2 |
| 28  (S) | 28  (S) | 34  (S) | 0  (R) | 30  (S) | S.spp.B3 |
| 24  (S) | 30  (S) | 35  (S) | 34  (S) | 27  (S) | S.spp.B4 |
| 25  (S) | 32  (S) | 36  (S) | 37  (S) | 28  (S) | STA2 |
| 24  (S) | 36  (S) | 33  (S) | 36  (S) | 30  (S) | STA3 |
| 26  (S) | 30  (S) | 35  (S) | 29  (S) | 23  (S) | STA4 |
| 25  (S) | 31  (S) | 36  (S) | 32  (S) | 26  (S) | S.spp.D1 |
| 29  (S) | 29  (S) | 34  (S) | 31  (S) | 21  (S) | S.spp.D2 |
| 25  (S) | 31  (S) | 32  (S) | 35  (S) | 25  (S) | S.spp.D3 |
| 26  (S) | 30  (S) | 34  (S) | 34  (S) | 24  (S) | S.spp.D4 |

(continued)

| **KAN** | **CFX** | **LVE** | **CRO** | **AMP** | *Salmonella* isolates |
| --- | --- | --- | --- | --- | --- |
| 22  (S) | 28  (S) | 36  (S) | 33  (S) | 29  (S) | 228 |
| 24  (S) | 29  (S) | 35  (S) | 31  (S) | 27  (S) | 875 |
| 28  (S) | 4  (S) | 30  (S) | 37  (S) | 28  (S) | 883 |
| 22  (S) | 35  (S) | 28  (S) | 36  (S) | 24  (S) | 1425 |
| 26  (S) | 32  (S) | 31  (S) | 34  (S) | 30  (S) | 1659 |
| 25  (S) | 4  (S) | 33  (S) | 40  (S) | 23  (S) | 2085 |
| 27  (S) | 4  (S) | 29  (S) | 32  (S) | 28  (S) | 2156 |
| 24  (S) | 2  (S) | 32  (S) | 29  (S) | 24  (S) | 2338 |
| 28  (S) | 32  (S) | 35  (S) | 33  (S) | 30  (S) | 2435 |
| 29  (S) | 33  (S) | 30  (S) | 37  (S) | 30  (S) | 2526 |
| 30  (S) | 33  (S) | 30  (S) | 40  (S) | 30  (S) | 2911 |
| 26  (S) | 4  (S) | 27  (S) | 30  (S) | 27  (S) | 3644 |
| 27  (S) | 43  (S) | 30  (S) | 37  (S) | 8  (R) | 4069 |
| 25  (S) | 30  (S) | 36  (S) | 32  (S) | 30  (S) | 4174 |
| 25  (S) | 31  (S) | 35  (S) | 36  (S) | 30  (S) | 4259 |

**MIC- Zone inhibition mm（susceptibility result):**

| **CHL** | **SXT** | **GEN** | **FOT** | **FOX** | **NAL** | **CIP** | **TET** | *Salmonella* isolates |
| --- | --- | --- | --- | --- | --- | --- | --- | --- |
| 8  (S) | 0.06/1.19  (S) | 1  (S) | 0.12  (S) | 4  (S) | 4  (S) | 0.03  (S) | 2  (S) | STC2 |
| 8  (S) | 0.06/1.19  (S) | 1  (S) | 0.06  (S) | 2  (S) | 4  (S) | 0.03  (S) | 2  (S) | STC3 |
| 16  (I) | 0.06/1.19  (S) | 0.5  (S) | 0.12  (S) | 4  (S) | 8  (S) | 0.06  (S) | 4  (S) | S.spp.B1 |
| 16  (I) | 0.12/2.38  (S) | 2  (S) | 0.25  (S) | 4  (S) | 1  (S) | 0.015  (S) | 4  (S) | S.spp.B2 |
| 16  (I) | 0.06/1.19  (S) | 0.5  (S) | 0.12  (S) | 4  (S) | 8  (S) | 0.03  (S) | 4  (S) | S.spp.B3 |
| 16  (I) | 0.06/1.19  (S) | 0.5  (S) | 0.12  (S) | 4  (S) | 4  (S) | 0.03  (S) | 4  (S) | S.spp.B4 |
| 16  (I) | 0.06/1.19  (S) | 0.5  (S) | 0.12  (S) | 4  (S) | 8  (S) | 0.03  (S) | 4  (S) | STA2 |
| 8  (S) | 0.06/1.19  (S) | 1  (S) | 0.06  (S) | 2  (S) | 4  (S) | 0.03  (S) | 4  (S) | STA3 |
| 8  (S) | 0.06/1.19  (S) | 1  (S) | 0.12  (S) | 4  (S) | 4  (S) | 0.03  (S) | 4  (S) | STA4 |
| 16  (I) | 0.06/1.19  (S) | 0.5  (S) | 0.12  (S) | 4  (S) | 8  (S) | 0.06  (S) | 4  (S) | S.spp.D1 |
| 16  (I) | 0.06/1.19  (S) | 1  (S) | 0.25  (S) | 8  (S) | 8  (S) | 0.06  (S) | 4  (S) | S.spp.D2 |
| 16  (I) | 0.06/1.19  (S) | 1  (S) | 0.12  (S) | 4  (S) | 8  (S) | 0.03  (S) | 4  (S) | S.spp.D3 |
| 16  (I) | 0.06/1.19  (S) | 0.5  (S) | 0.25  (S) | 4  (S) | 8  (S) | 0.03  (S) | 4  (S) | S.spp.D4 |

(Continued)

| **CHL** | **SXT** | **GEN** | **FOT** | **FOX** | **NAL** | **CIP** | **TET** | *Salmonella* isolates |
| --- | --- | --- | --- | --- | --- | --- | --- | --- |
| 8  (S) | 0.03/0.59  (S) | 0.5  (S) | 0.25  (S) | 4  (S) | 4  (S) | 0.015  (S) | 2  (S) | 228 |
| 8  (S) | 0.06/1.19  (S) | 0.5  (S) | 0.06  (S) | 4  (S) | 4  (S) | 0.015  (S) | 2  (S) | 875 |
| 8  (S) | 0.12/2.38  (S) | 0.5  (S) | 0.12  (S) | 4  (S) | 8  (S) | 0.03  (S) | 4  (S) | 883 |
| 16  (I) | 0.12/2.38  (S) | 1  (S) | 0.25  (S) | 4  (S) | 4  (S) | 0.03  (S) | 4  (S) | 1425 |
| 8  (S) | 0.12/2.38  (S) | 0.5  (S) | 0.12  (S) | 4  (S) | >128  (R) | 0.25  (I) | 2  (S) | 1659 |
| 8  (S) | 0.12/2.38  (S) | 1  (S) | 0.12  (S) | 4  (S) | 2  (S) | 0.015  (S) | 2  (S) | 2085 |
| 16  (I) | 0.12/2.38  (S) | 0.5  (S) | 0.06  (S) | 4  (S) | 8  (S) | 0.03  (S) | >64  (R) | 2156 |
| 8  (S) | 0.06/1.19  (S) | 0.5  (S) | 0.12  (S) | 4  (S) | >128  (R) | 0.25  (I) | 64  (R) | 2338 |
| 8  (S) | 0.06/1.19  (S) | 0.5  (S) | 0.12  (S) | 4  (S) | 4  (S) | 0.03  (S) | 4  (S) | 2435 |
| 8  (S) | <0.03/0.59  (S) | 0.5  (S) | 0.06  (S) | 4  (S) | >128  (R) | 0.5  (I) | >64  (R) | 2526 |
| 4  (S) | 0.12/2.38  (S) | 0.5  (S) | 0.06  (S) | 2  (S) | >128  (R) | 0.25  (I) | 1  (S) | 2911 |
| 8  (S) | 0.03/0.59  (S) | 0.5  (S) | 0.25  (S) | 4  (S) | 4  (S) | 0.03  (S) | 2  (S) | 3644 |
| 8  (S) | ≥32/608  (R) | 0.25  (S) | 0.25  (S) | 8  (S) | >128  (R) | 0.5  (I) | 4  (S) | 4069 |
| 16  (I) | 0.12/2.38  (S) | 1  (S) | 0.25  (S) | 4  (S) | 4  (S) | 0.03  (S) | 4  (S) | 4174 |
| 8  (S) | 0.12/2.38  (S) | 0.5  (S) | 0.5  (S) | 32  (R) | 4  (S) | 0.015  (S) | >64  (R) | 4259 |

**Abbreviations: AMP:** Ampicillin**,** , **CRO:** Ceftriaxone**, LVE:** Levofloxacin **CFX:** Cefixime**,**

**K:** kanamycine, **TET:** Tetracycline, **CIP:** Ciprofloxacin**, NAL:** Naladixic acid**, FOX:** Cefoxitin,

**FOT:** Cefotaxime, **GEN:** gentamicn, **SXT:** Trimethoprim/sulfamethoxazole**, CHL:** Chloramphenicol.
