# Supplementary figures and images for: Identification of Salmonella Bredeney Resistant to Third-Generation Cephalosporins in Saudi Arabia
Source: Front Cell Infect Microbiol. 2019 Nov 20;9:390. doi: 10.3389/fcimb.2019.00390 (PMC6879462; doi:10.3389/fcimb.2019.00390)

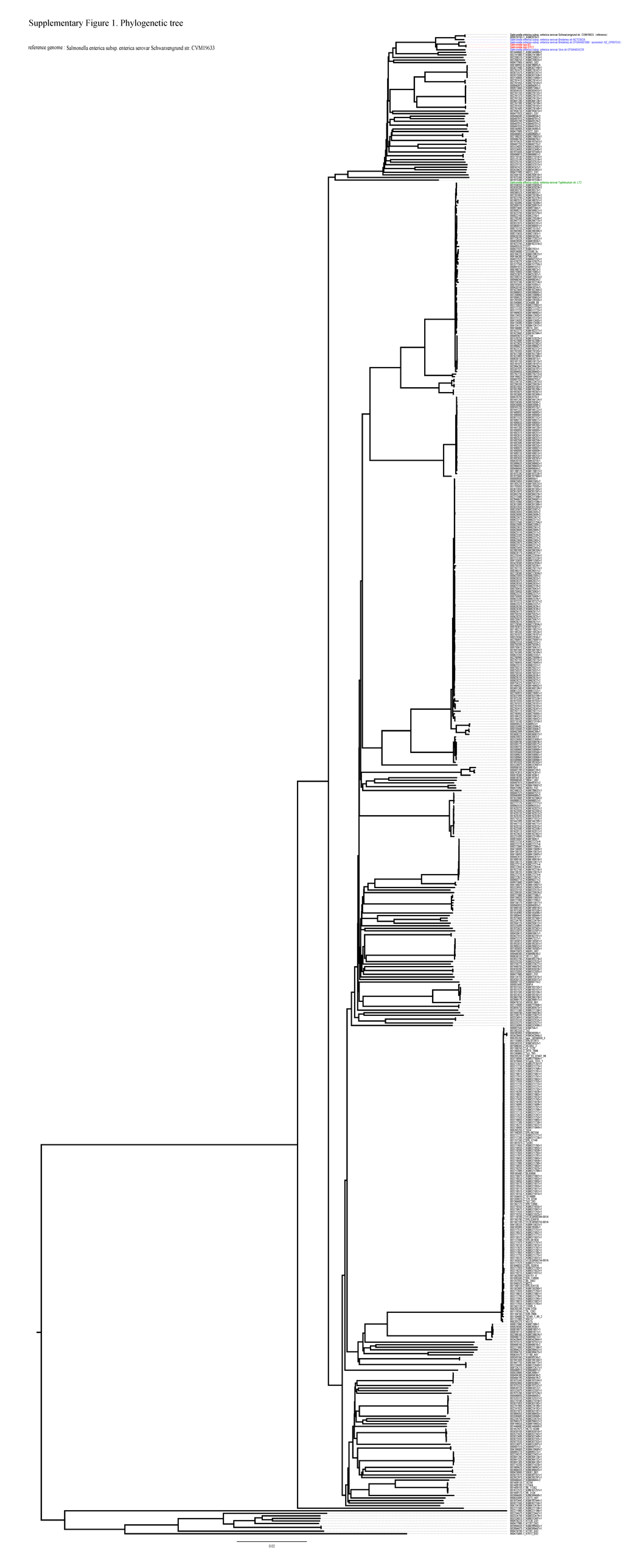

Supplement: Supplementary file 9 [file Image_1.tif]
